# Report of Two Cases of Traumatic Tetanus, with Recovery

**Published:** 1882-10

**Authors:** F. W. Stebalts

**Affiliations:** 802 S. Halsted St., Chicago


### ARTICLE V.

### REPORT OF TWO CASES OF TRAUMATIC TETANUS, WITH RECOVERY. By F. W. STEBALTS, M.D.

CASE I.—July 16 of the current year, I was summoned to attend H., a boy thirteen years old, who was complaining of stiffness of the whole body. He had trismus to such a degree that only a little fluid could be swallowed, the jaws opening one quarter inch. The neck was stiff and swollen. There was a slight rise in the temperature, restlessness, sleeplessness and constipation. Upon inquiry it was found that the patient had shot himself in the hand with a toy pistol the 4th of July. The wound had subsequently healed. On July 14 he fell into a ditch full of water and got drenched. Tetanus supervened in the next few days.

At first, looking upon the case as one of rheumatic tetanus, I prescribed ten grain doses of salicylate of sodium, to be taken every two hours. I also ordered effervescent citrate of magnesia to move the bowels.

July 18, the bowels had moved, the patient was improved so that he could open the mouth much wider, and he could swallow tolerably well. The salicylate was continued, and the bromide of potassium was given at night for the restlessness. A few days later the patient was able to be out, and subsequently entirely recovered.

CASE II.—July 18, I was called to attend V., a girl fifteen years of age, who for three days had had stiffness of the neck, difficulty in opening the mouth more than a few lines, inability to move, and difficulty in swallowing even liquids. I was called just as spasms of all the muscles were setting in, the spasms being more marked in the muscles of the neck. There was constipation, fever and sleeplessness. Upon examination, it was found that the patient had been shot in the palm of the hand on July 4th, and the wound, which had not completely healed, was marked by a large cicatrix.

The treatment in this case consisted of citrate of magnesia, which moved the bowels after two days. She took ten grains of chloral and fifteen grains of bromide of potassium every three hours. This produced sleep. After a couple of days the jaws could be opened half an inch, and the general stiffness had slightly diminished. I then added one-fourth gr. of the solid extract of belladonna to the preceding treatment. On the third day, I added tonic doses of quinine, which was discontinued after two or three days, and meanwhile the fever had disappeared.

A week after the beginning of the treatment, the patient could open her mouth an inch wide at times, but the spasms sometimes recurred, when the trismus was more marked, and also the stiffness of the body. Motion returned in the arms after fourteen days, and then in the legs, so that she could be up around, although there was yet difficulty in opening the mouth. I now added the extract of cannabis indica, one-fourth grain, to the bromides, and gave the two three times a day.

After she was able to be out, that is, in the first week of August, I gave the elixir of quinine, iron and strychnia three times a day, and a month after the beginning of the sickness the patient was entirely well.

802 *S. Halsted St., Chicago.*

---

RUSH MEDICAL COLLEGE opened its session Tuesday, September 26, 1882, with an introductory address delivered by Prof. Norman Bridge. The building has been thoroughly renovated since its last session. They have matriculated four hundred and seventy students.

WE are pleased to learn that Dr. Edw. W. Jenks, who recently established a gynæcological hospital at Geneva, Illinois, is meeting with good success He finds the building entirely inadequate for his purposes, and is making additions thereto.

